# Use of Observation Care in US Emergency Departments, 2001 to 2008

**DOI:** 10.1371/journal.pone.0024326

**Published:** 2011-09-14

**Authors:** Arjun K. Venkatesh, Benjamin P. Geisler, Jennifer J. Gibson Chambers, Christopher W. Baugh, J. Stephen Bohan, Jeremiah D. Schuur

**Affiliations:** 1 Brigham and Women's Hospital-Massachusetts General Hospital-Harvard Affiliated Emergency Medicine Residency, Boston, Massachusetts, United States of America; 2 Institute for Technology Assessment, Massachusetts General Hospital, Harvard Medical School, Boston, Massachusetts, United States of America; 3 University of New England College of Osteopathic Medicine, Biddeford, Maine, United States of America; 4 Department of Emergency Medicine, Brigham and Women's Hospital, Boston, Massachusetts, United States of America; 5 Division of Emergency Medicine, Harvard Medical School, Boston, Massachusetts, United States of America; Yale University School of Medicine, United States of America

## Abstract

**Background:**

Observation care is a core component of emergency care delivery, yet, the prevalence of emergency department (ED) observation units (OUs) and use of observation care after ED visits is unknown. Our objective was to describe the 1) prevalence of OUs in United States (US) hospitals, 2) clinical conditions most frequently evaluated with observation, and 3) patient and hospital characteristics associated with use of observation.

**Methods:**

Retrospective analysis of the proportion of hospitals with dedicated OUs and patient disposition after ED visit (discharge, inpatient admission or observation evaluation) using the National Hospital Ambulatory Medical Care Survey (NHAMCS) from 2001 to 2008. NHAMCS is an annual, national probability sample of ED visits to US hospitals conducted by the Center for Disease Control and Prevention. Logistic regression was used to assess hospital-level predictors of OU presence and polytomous logistic regression was used for patient-level predictors of visit disposition, each adjusted for multi-level sampling data. OU analysis was limited to 2007–2008.

**Results:**

In 2007–2008, 34.1% of all EDs had a dedicated OU, of which 56.1% were under ED administrative control (EDOU). Between 2001 and 2008, ED visits resulting in a disposition to observation increased from 642,000 (0.60% of ED visits) to 2,318,000 (1.87%, p<.05). Chest pain was the most common reason for ED visit resulting in observation and the most common observation discharge diagnosis (19.1% and 17.1% of observation evaluations, respectively). In hospital-level adjusted analysis, hospital ownership status (non-profit or government), non-teaching status, and longer ED length of visit (>3.6 h) were predictive of OU presence. After patient-level adjustment, EDOU presence was associated with increased disposition to observation (OR 2.19).

**Conclusions:**

One-third of US hospitals have dedicated OUs and observation care is increasingly used for a range of clinical conditions. Further research is warranted to understand the quality, cost and efficiency of observation care.

## Introduction

Over the last decade, emergency department (ED) use has increased while the number of inpatient hospital beds has decreased presenting a bottleneck for patients in need of acute care services.[1], [2] The Center for Disease Control (CDC) estimated 124 million ED visits in 2008, which represents an increase of more than 20% in the last decade despite the closure of 9% of EDs and nearly 200,000 hospital beds. [1]–[3] As over half of all hospital admissions now originate in the ED, this service pattern has contributed to crowding in the ED - the primary entry point to acute care services.[4], [5] Coincidentally, observation care, which utilizes rapid diagnostic and treatment protocols, has grown as an alternative to ”short-stay” inpatient admissions [6] Numerous studies have demonstrated that protocol-driven observation care can deliver equivalent clinical outcomes at lower costs and shorter lengths of stay for many conditions including: chest pain, syncope, atrial fibrillation, asthma, and transient ischemic attack.[6], [7]

There has been limited study of observation services and utilization at the national level despite increasing policy attention from the Center for Medicare and Medicaid Services (CMS). In 2006 CMS initiated the Recovery Audit Contractor (RAC) program with the aim of identifying potential waste in the Medicare program, and subsequently short stay hospital admissions, which were deemed to occur in the “wrong setting”, became a primary target for charge recovery. Subsequently hospitals have shifted to billing patients for observation services rather than inpatient care for short stays.[8]–[10] This policy change combined with expanded CMS reimbursement for observation services [11] may have impacted the use of observation evaluations. Furthermore, there has been no recent study of the national capacity to deliver observation care as the only estimate of the number of observation units (OUs) is derived from a 2003 survey of 522 hospitals, which reported that 19 percent of hospitals had a dedicated OU and an additional 12 percent planned to open an OU.[12] The 2003 survey reported ED directors' impression that the five most common conditions observed in an OU as chest pain, abdominal pain, asthma, “general medical ailments” and dehydration.

We analyzed data from the National Hospital Ambulatory Medical Care Survey (NHAMCS) from 2001 to 2008 in order to 1) describe the prevalence of dedicated EDOUs and hospital observation units (HOUs) in US hospitals, 2) describe the clinical conditions most frequently evaluated with observation services after ED visits and 3) describe patient and hospital characteristics that are associated with use of observation services after ED visits. We hypothesized that the proportion of ED patients receiving observation services has increased over time, and that the relative frequency of ED patients placed in observation will be higher in hospitals with dedicated OUs.

## Methods

### Study Design

We performed a retrospective analysis of a nationally representative survey of EDs and ED visits from 2001 to 2008.

### Study Dataset

The National Hospital Ambulatory Medical Care Survey (NHAMCS) is an annual, four-stage, national probability sample of ambulatory and ED visits to non-institutional general and short-stay hospitals located in the US, excluding Federal, military, and Veterans Administrations hospitals, conducted by the Center for Disease Control and Prevention's Division of Health Care Statistics (NCHS). NHAMCS is structured to cover geographic primary sampling units, hospitals within these sampling units, EDs within these hospitals, and patients within these EDs. NHAMCS defines a hospital with an ED as providing emergency services 24 hours a day either at this hospital or elsewhere.[13]

We analyzed data from 2001 to 2008 on the ED component of the survey which samples approximately 400 EDs each year and provides a nationally representative sample of ED and hospital use. Sample estimates are weighted based on survey sampling probabilities to provide national estimates. The methods have been described previously.[13]

The survey is administered by ED staff that are provided training, educational material, and data collection tools by trained field representatives from the U.S. Census Bureau. The staff complete patient record forms for a systematically random sample of patient visits during a randomly assigned 4-week reporting period. Data obtained include patient demographics, insurance status, patient complaints, services provided, and patient disposition. The patient record form is completed at or near the time of visit for each sampled patient. Additionally, each participating hospital completes a survey about hospital and ED characteristics at the beginning of the sampling period. In 2007 and 2008 this survey included questions asking, “Does your ED have an observation or clinical decision unit?” and “Is your observation or clinical decision unit administratively a part of the ED or the inpatient side of the hospital?” We define OUs that are “administratively part of the ED” as EDOUs, while we defined OUs that are “administratively part of the inpatient side of the hospital” as hospital observation units (HOUs).

The institutional review board of our hospital has exempted analyses of the NHAMCS public dataset from review, as it contains no patient identifiers.

### Data Collection and Processing

Hospital-level characteristics assigned by NCHS to each record include: presence of a dedicated OU, administrative control of the OU (EDOU or HOU), ownership status (nonprofit, state or local government, or proprietary), geographic region (Northeast, South, Midwest or West), urban location (Metropolitan statistical area [MSA] or non-MSA).[13] We calculated additional hospital-level variables by analyzing patient visits for each hospital in the sample. Teaching status was defined as academic for hospitals in which a resident or intern saw at least one ED patient. Waiting time was averaged across patient records within each hospital and then grouped in quintiles. Hospital socioeconomic measures were grouped in quintiles and included: percent of uninsured or underinsured ED patients (primary payment sources of self, charity, Medicaid/SCHIP) and percent of ED visits by Black patients.

Patient-level data elements included standard demographic information as well as reasons for visit, triage acuity, previous visits, discharge diagnosis codes, and visit disposition. Patient's chief complaints were categorized by organ system and defined by the *Reason for Visit (RFV) for Ambulatory Care Coding* System.[14] We grouped ICD-9 discharge diagnosis codes into clinically distinct conditions using Clinical Classifications Software (CCS). The CCS for ICD-9-CM is a validated diagnosis and procedure categorization scheme that is updated yearly, and has been used in a variety of healthcare services studies.[15] The RFV coding system is distinct from the CCS coding system since it represents the patient prior to evaluation described by a non-mutually exclusive set of chief complaints, whereas the CCS code is determined based on the discharge diagnosis applied to a patient's visit after evaluation.

### Outcomes Measures

The primary hospital outcome was proportion of EDs with dedicated OUs. The primary patient outcome was disposition after ED visit. We categorized the disposition of patients at the end of the ED visit as discharged to home, admitted to inpatient care or assigned to observation status. The ultimate disposition of patients placed observation status was categorized as discharge to home or admit to inpatient status. We calculated several metrics to quantify the use of observation relative to other dispositions. The *ED observation proportion* is the proportion of all ED visits with an initial disposition to observation. The *ED admission proportion* is the proportion of all ED visits with an initial disposition to inpatient admission. The *admission from observation proportion* is the proportion of ED observation visits who are subsequently admitted to inpatient status. The *observation to admission ratio* is number of ED visits followed by observation divided by the number of ED visits followed by observation or inpatient admissions. We calculated the association between observation unit (EDOU or HOU) presence and disposition to observation compared to discharge or admission. Explanatory variables included hospital, patient and visit characteristics detailed above.

### Data Analysis

The number of ED visits with subsequent observation services was counted by year compared to discharged and admitted patients. The most frequent reasons for visit (RFV) and CCS codes leading to observations were counted separately. Logistic regression was performed to obtain unadjusted and adjusted ORs and 95% confidence intervals (CI) of hospital-level variables associated with OU presence. Polytomous logistic regression was performed on the patient level data, comparing the odds of being initially observed versus being discharged or being admitted without being observed. All variables that were significant in the bivariate analysis were included in the multivariate models. All data analyses were conducted using SAS 9.2 (SAS Institute, Cary, NC), accounting for the complex survey design with appropriate weighting.[16]

## Results

In 2007-2008 34.5% (95%CI: 27.95%–41.15%) of the hospitals with EDs reported having an OU. Of the hospitals with OUs 56.5% (95%CI: 47.34%–65.57%) reported that they were EDOUs, while 35.6% (95%CI: 26.64%–44.62%) were HOUs and 3.9% (95%CI: 0.39%–7.48%) unknown. Neither the proportion of hospitals with OUs, nor the proportion of OUs's administratively run by the ED changed between 2007 and 2008. The hospital characteristics associated with the presence of a dedicated OU included ownership status (non-proprietary and government, non-Federal), non-teaching status and hospitals with longer average ED length of visit. **(** **Table 1** **)**

**Table 1:** Hospital level predictors of dedicated Observation Unit (OU) presence, 2007–2008.

Hospital Factor	%, hospitals with dedicated OU	Unadjusted OR (95%CI)	Adjusted OR (95% CI)
**Geographic Region**			
South	33.2%	RG	RG
Midwest	34.0%	1.04 (0.49, 2.20)	1.32 (0.65, 2.70)
Northeast	34.4%	1.07 (0.56, 2.03)	1.12 (0.52, 2.42)
West	35.9%	1.12 (0.45, 2.81)	1.14 (0.44, 2.91)
**Urban Status**			
non-MSA	27.7%	RG	RG
MSA	37.5%	1.57 (0.79, 3.10)	1.28 (0.55, 2.96)
**Ownership Status**			
Proprietary	19.2%	RG	RG
Voluntary, non-profit	33.8%	2.15 (1.11, 4.13)	2.42 (1.17, 5.00)
Government, non-Federal	48.9%	4.02 (1.73, 9.36)	4.31 (1.62, 11.46)
**Teaching Status**			
Teaching ED	31.8%	RG	RG
non-Teching	35.5%	1.17 (0.79, 1.75)	1.70 (1.06, 2.74)
**Uninsured or Underinsured, Quintile** *			
1st (0%–26%)	29.5%	RG	RG
2nd (26%–38%)	25.4%	0.82 (0.35, 1.92)	0.98 (0.38, 2.51)
3rd (38%–48%)	41.2%	1.68 (0.85, 3.30)	1.90 (0.93, 3.89)
4th (48%–62%)	32.8%	1.17 (0.49, 2.80)	1.57 (0.62, 4.00)
5th (62%–99%)	47.0%	2.13 (0.91, 4.95)	2.24 (0.92, 5.44)
**Race (%Black), Quintile**			
1st (0%–3%)	28.5%	RG	RG
2nd (3%–11%)	33.6%	1.27 (0.65, 2.48)	1.23 (0.52, 2.93)
3rd (11%–22%)	45.7%	2.12 (1.11, 4.05)	1.56 (0.74, 3.30)
4th (22%–40%)	34.8%	1.36 (0.62, 2.99)	1.02 (0.41, 2.58)
5th (40%–96%)	34.1%	1.30 (0.64, 2.66)	0.85 (0.41, 1.77)
**Length of ED Visit (Hours), Quintile**			
1st (0.9–2.4)	29.40%	RG	RG
2nd (2.4–3.0)	26.70%	0.87 (0.45, 1.69)	0.86 (0.41, 1.82)
3rd (3.0–3.6)	38.10%	1.48 (0.78, 2.81)	1.64 (0.73, 3.67)
4th (3.6–4.3)	45.90%	2.03 (1.04, 3.97)	2.40 (1.06, 5.45)
5th (4.3–13.5)	52.90%	2.69 (1.28, 5.65)	3.03 (1.26, 7.27)

*Uninsured and Underinsured includes all Medicaid, Self-Pay and Charity Care.

Over the seven year study period there were 287,803 NHMACS records available for analysis representing more than 910 million ED visits (**Table 2**). Emergency department visits increased from 107 million in 2001 to 123 million in 2008. ED visits with subsequent observation care increased from 642,000 in 2001 (0.60% of all ED visits, 95%CI: 0.43–0.76%) in 2001 to 2,318,000 in 2008 (1.87% of all ED visits, 95%CI: 1.49%–2.26%%). Over that time period, the percentage of observation visits subsequently admitted to the inpatient status increased from 22,000 (3.45% of all observation evaluations, 95%CI: 0.00–7.33%) to 761,000 (32.81% of all observation evaluations, 95%CI: 25.67–39.94%%). Patients admitted for inpatient stay after observation represented 4.6% of all inpatient admissions in 2008, a more than 23-fold relative increase compared to 2001.

**Table 2:** National Estimates of ED Disposition, 2001-2008‡.

Year	ED visits	Observation evaluations	Inpatient Admissions	Inpatient Admissions Following Observation	% of Inpatient Admissions from Observation
	n	*n (% of all ED Visits* * *)*	*n (% of all ED Visits* ** *)*	*n (% of all Observations* † *)*	%
**2001**	107,490,000	642,000 (0.60%)	12,626,000 (11.75%)	22,000 (3.46%)	0.18%
**2002**	110,155,000	688,000 (0.62%)	13,471,000 (12.23%)	40,000 (5.76%)	0.29%
**2003**	113,903,000	384,000 (0.34%)	15,809,000 (13.88%)	56,000 (14.64%)	0.36%
**2004**	110,216,000	613,000 (0.56%)	14,618,000 (13.26%)	151,000 (24.64%)	1.03%
**2005**	115,323,000	1,010,000 (0.88%)	13,867,000 (12.02%)	217,000 (21.46%)	1.56%
**2006**	119,192,000	1,265,000 (1.06%)	15,207,000(12.76%)	346,000 (27.39%)	2.28%
**2007**	116,802,000	2,452,000 (2.10%)	14,639,000 (12.53%)	448,000 (18.25%)	3.06%
**2008**	123,761,000	2,318,000 (1.87%)	16,570,000 (13.39%)	761,000 (32.81%)	4.59%
**Total**	916,842,000	9,372,000 (1.02%)	116,807,000 (12.74%)	2,040,000 (21.77%)	1.30%

*This is the *ED observation proportion,* the proportion of all ED visits with a disposition to observation.

**This is the *ED admission proportion*, the proportion of all ED visits with a disposition to inpatient admission.

†This is the *admission from observation proportion;* the proportion of ED observation visits subsequently admitted to inpatient status.

‡All visit counts were rounded to the closest thousand, and all percentages are based on weighted frequencies prior to rounding.

The most common reason for visit (RFV) for patient subsequently placed under observation was chest pain **Table 3** shows the top 10 RFV for patients assigned to observation care at the end of the ED visit including the percentage of all episodes of observation care attributed to that RFV. The top 10 RFVs accounted for 44.1% of all observation use. Of the ten most common RFVs, the RFV with the highest frequency of subsequent observation care was “Chest pain” for which 1,786,000 (4.08% of all ED visits, 95%CI: 3.32%–4.85%) of visits were observed, while “Chest discomfort, pressure, tightness” was the RFV with the highest observation proportion (4.88%, 95%CI: 2.69%–7.07%). “Fainting” was the ED RFV with the highest likelihood of subsequent inpatient admission following observation care (34.3% of all “fainting” evaluations, 95%CI: 16.8–51.7. When comparing the relative use of observation to inpatient admission, chest pain had the highest relative proportion as 17.8% of patients with post-ED evaluations for chest pain were dispositioned to observation services.

**Table 3:** Top 10 Reasons for Visit and Discharge Diagnoses for Patients evaluated in Observation, 2001–2008.

Top 10 Reasons for Visits for Patient Subsequently Evaluated in Observation, 2001–2008‡										
Reason for Visit (RFV)		ED Visits, *n*	Observation Evaluations, *n*	% of all Observation Evaluations (95% CI)	ED Observation Proportion (95% CI)*	Inpatient Admissions, *n*	% of all Inpatient Admissions (95% CI)	ED Admission Proportion (95% CI)**	Observation to Admission Ratio¥	Admission from Observation Proportion† (95% CI)
1	Chest pain	43,732,000	1,786,000	19.06% (16.80%, 21.31%)	4.08% (3.32%, 4.85%)	13,540,000	11.80% (11.30%, 12.29%)	30.96% (29.26%, 32.66%)	11.7%	20.99% (13.91%, 28.07%)
2	Abdominal pain, cramps, spasms, NOS	44,006,000	571,000	6.09% (4.87%, 7.30%)	1.30% (1.00%, 1.59%)	8,224,000	7.17% (6.72%, 7.62%)	18.69% (17.60%, 19.78%)	6.5%	17.48% (8.74%, 26.21%)
3	Shortness of breath	23,232,000	433,000	4.62% (3.62%, 5.61%)	1.86% (1.37%, 2.36%)	9,122,000	7.95% (7.52%, 8.38%)	39.26% (37.31%, 41.21%)	4.5%	28.25% (15.36%, 41.15%)
4	Nausea	12,704,000	215,000	2.30% (1.66%, 2.94%)	1.69% (1.17%, 2.22%)	2,570,000	2.24% (2.01%, 2.47%)	20.23% (18.24%, 22.22%)	7.7%	24.44% (8.01%, 40.87%)
5	Fainting (syncope)	6,223,000	209,000	2.23% (1.57%, 2.89%)	3.36% (2.29%, 4.43%)	1,754,000	1.53% (1.36%, 1.70%)	28.19% (25.55%, 30.83%)	10.6%	34.25% (16.76%, 51.74%)
6	Vertigo and dizziness	12,398,000	208,000	2.22% (1.54%, 2.91%)	1.68% (1.13%, 2.23%)	2,216,000	1.93% (1.74%, 2.12%)	17.87% (16.13%, 19.62%)	8.6%	26.76% (13.98%, 39.54%)
7	Other Psychiatric Symptoms	7,208,000	204,000	2.18% (1.37%, 2.98%)	2.83% (1.68%, 3.98%)	2,598,000	2.26% (2.03%, 2.50%)	36.05% (32.93%, 39.17%)	7.3%	22.95% (6.54%, 39.37%)
8	Vomiting	20,034,000	194,000	2.07% (1.39%, 2.75%)	0.97% (0.65%, 1.29%)	2,671,000	2.33% (2.09%, 2.57%)	13.33% (11.84%, 14.82%)	6.8%	17.24% (3.28%, 31.20%)
9	Labored or difficult breathing (dyspnea)	11,736,000	190,000	2.02% (1.35%, 2.69%)	1.62% (1.03%, 2.20%)	3,513,000	3.06% (2.75%, 3.38%)	29.93% (27.74%, 32.13%)	5.1%	15.18% (0.21%, 30.15%)
10	Chest discomfort, pressure, tightness	3,823,000	186,000	1.99% (1.09%, 2.89%)	4.88% (2.69%, 7.07%)	1,107,000	0.96% (0.83%, 1.10%)	28.96% (25.73%, 32.20%)	14.4%	8.17% (1.49%, 14.85%)

Top 10 Diagnoses for ED Visits Subsequently Admitted to Observation, 2001–2008‡										
Clinical Diagnosis (CCS Category$)		ED Visits, *n*	Observation Evaluations *n*	% of all Observation Evaluations (95% CI)	ED Observation Proportion (95% CI)*	Inpatient Admissions, *n*	% of all Inpatient Admissions (95% CI)	ED Admission Proportion (95% CI)**	Observation to Admission Ratio¥	Admission from Observation Proportion† (95% CI)
1	Nonspecific chest pain	28,193,000	1,601,000	17.08% (14.49%, 19.68%)	5.68% (4.53%, 6.83%)	10,002,000	8.72% (8.17%, 9.26%)	35.48% (33.26%, 37.69%)	13.8%	15.87% (9.19%, 22.55%)
2	Abdominal pain	36,388,000	435,000	4.64% (3.71%, 5.57%)	1.19% (0.92%, 1.57%)	5,183,000	4.52% (4.17%, 4.87%)	14.24% (13.08%, 15.41%)	7.7%	13.83% (6.59%, 21.07%)
3	Syncope	7,874,000	259,000	2.76% (2.00%, 3.52%)	3.29% (2.32%, 4.35%)	2,684,000	2.34% (2.11%, 2.56%)	34.09% (31.51%, 36.67%)	8.8%	29.93% (14.37%, 45.49%)
4	Cardiac dysrhythmias	7,901,000	245,000	2.62% (1.75%, 3.49%)	3.11% (1.98%, 4.23%)	2,544,000	2.22% (2.02%, 2.41%)	32.20% (29.66%, 34.74%)	8.8%	17.95% (4.25%, 31.65%)
5	Mood disorders	7,481,000	235,000	2.50% (1.64%, 3.37%)	3.14% (1.98%, 4.29%)	1,784,000	1.55% (1.36%, 1.75%)	23.85% (21.24%, 26.45%)	11.6%	10.42% (3.11%, 17.73%)
6	Skin and subcutaneous tissue infections	20,265,000	206,000	2.20% (1.44%, 2.96%)	1.02% (0.64%, 1.40%)	2,416,000	2.10% (1.91%, 2.30%)	11.92% (10.64%, 13.20%)	7.9%	29.71% (13.62%, 45.80%)
7	Congestive heart failure; nonhypertensive	5,601,000	168,000	1.79% (1.16%, 2.42%)	3.00% (1.87%, 4.12%)	4,109,000	3.58% (3.30%, 3.86%)	73.36% (70.64%, 76.09%)	3.9%	29.97% (11.63%, 48.30%)
8	Coronary atherosclerosis and heart disease	3,745,000	163,000	1.74% (1.10%, 2.38%)	4.36% (2.70%, 6.02%)	2,563,000	2.23% (2.00%, 2.47%)	68.42% (64.37%, 72.48%)	6.0%	19.42% (2.76%, 36.08%)
9	Other injuries due to external causes	24,800,000	159,000	1.70% (1.13%, 2.27%)	0.64% (0.42%, 0.87%)	1,930,000	1.68% (1.37%, 1.99%)	7.78% (6.29%, 9.27%)	7.6%	16.81% (5.87%, 27.75%)
10	Transient cerebral ischemia	1,981,000	150,000	1.60% (0.56%, 2.64%)	7.57% (2.99%, 12.16%)	9,784,000	0.85% (0.73%, 0.98%)	49.38% (43.84%, 54.91%)	13.3%	14.20% (0.00%, 29.56%)

‡All visit counts were rounded to the closest thousand.

*This is the *ED observation proportion,* the proportion of all ED visits with a disposition to observation.

**This is the *ED admission proportion: the* proportion of patients admitted for inpatient services of all ED patients.

†This is the *admission from observation proportion;* the proportion of ED observation visits subsequently admitted to inpatient status.

¥ This is the Observation to Admission Ratio: the ratio of all ED visits with an initial disposition to observation to all patients with a disposition to observation or inpatient admission.

$ Clinical Classificatino System (16)

The most common ED discharge CCS condition for patients placed in observation was nonspecific chest pain (17.1% of all observation evaluations, 95% CI: 14.5% to 19.7%). The 10 most common ED discharge diagnosis CCS accounted for 39.9% of all observation use (**Table 3**). Transient cerebral ischemia had the highest observation proportion at 7.6% (95%CI: 3.0%–12.2%) of ED patients with this discharge diagnosis were initially dispositioned to observation. Congestive heart failure was the observation discharge diagnosis with the highest likelihood of subsequent inpatient admission following observation care (30.0% of all CHF observation evaluations, 95%CI: 11.6%–48.3%).

Patient characteristics and their relationship to subsequent observation evaluation are listed in **Table 4**. Patient characteristics associated with observation use compared to ED discharge include ambulance arrival, evaluation by resident or intern, and ED visit in the last 72 hours. Among patient characteristics associated with observation disposition compared to inpatient admission, the adjusted analysis only found significant association with evaluation by a Nurse Practitioner or PA and ED visit in the last 72 hours.

**Table 4:** Patient level predictors of observation care, 2001–2008.

	Initial Disposition following ED evaluation				
Predictor	Discharge	Observation	Inpatient Admission	Observation vs. Discharge	Observation vs. Inpatient
	%	%	%	OR (95% CI)	OR (95% CI)
**Patient Demographics**					
**Age**					
Under 15 years	22.49%	7.17%	6.00%	Reference	Reference
15–24 years	17.68%	7.90%	5.54%	1.42 (1.04, 1.94)	1.19 (0.85, 1.67)
25–44 years	30.77%	26.29%	18.07%	2.69 (2.00, 3.62)	1.23 (0.91, 1.67)
45–64 years	18.38%	29.55%	27.63%	4.54 (3.38, 6.10)	0.96 (0.71, 1.30)
65–74 years	4.71%	11.38%	14.39%	5.64 (3.89, 8.18)	0.81 (0.54, 1.22)
75 years and over	5.97%	17.71%	28.37%	5.12 (3.64, 7.20)	0.57 (0.39, 0.83)
**Gender**					
Male	45.95%	45.37%	46.51%	Reference	Reference
Female	54.05%	54.63%	53.49%	0.95 (0.85, 1.06)	1.09 (0.97, 1.23)
**Race**					
White, non-Hispanic	61.62%	66.47%	69.89%	Reference	Reference
Black, non-Hispanic	22.02%	19.40%	17.39%	0.89 (0.72, 1.10)	1.03 (0.84, 1.26)
Hispanic	13.37%	11.12%	9.81%	0.96 (0.73, 1.26)	1.01 (0.76, 1.33)
Other	3.00%	3.01%	2.91%	1.04 (0.69, 1.56)	1.09 (0.73, 1.62)
**Insurance Status**					
Private insurance	42.73%	34.23%	30.86%	Reference	Reference
Medicaid	25.49%	22.34%	16.76%	1.29 (1.02, 1.63)	1.22 (0.97, 1.54)
Medicare	12.76%	30.90%	43.50%	1.34 (1.07, 1.69)	0.93 (0.74, 1.17)
Self-pay	19.02%	12.53%	8.88%	0.82 (0.64, 1.04)	1.15 (0.90, 1.47)
**Patient Arrival Characteristics**					
**Arrived by ambulance**					
No	91.33%	71.45%	69.30%	Reference	Reference
Yes	8.67%	28.55%	30.70%	2.59 (2.20, 3.04)	1.05 (0.89, 1.24)
**Arrival from Nursing Home**					
No	97.78%	92.87%	90.37%	Reference	Reference
Yes	2.22%	7.13%	9.63%	1.15 (0.91, 1.47)	0.98 (0.77, 1.25)
**Time to Triage Assessment**					
Immediate-14 mins	24.35%	39.64%	45.13%	Reference	Reference
15–60 mins	38.69%	46.37%	40.81%	0.71 (0.60, 0.83)	1.15 (0.99, 1.33)
1–2 h	23.84%	11.03%	10.84%	0.33 (0.25, 0.42)	1.01 (0.77, 1.33)
2–24 h	13.12%	2.96%	3.22%	0.19 (0.11, 0.31)	0.97 (0.61, 1.53)
**ED Visit Characteristics**					
**Provider Type Seen**					
No Nurse Practitioner (NP)/Pysician Asst.(PA)	89.00%	90.02%	93.66%	Reference	Reference
Seen by NP/PA	11.00%	9.98%	6.34%	1.33 (0.94, 1.89)	1.73 (1.22, 2.46)
No Resident/Intern	91.96%	83.33%	84.47%	Reference	Reference
Seen by Resident/Intern	8.04%	16.67%	15.53%	2.62 (1.94, 3.53)	1.20 (0.91, 1.59)
**Previous Care/Revisitation**					
Index ED visit	96.28%	94.74%	96.29%	Reference	Reference
Seen in ED in last 72 hours	3.72%	5.26%	3.71%	1.51 (1.14, 1.99)	1.33 (1.01, 1.77)

*Chi-square of proportions.

**Polytomous logistic regression.

In 2007–2008, hospitals with an OU were more likely than hospitals without OUs to disposition patients to observation in comparison to discharge (OR for EDOU: 1.93, 95%CI: 1.27–2.60; OR for HOU: 1.75(1.11–2.76), but not more likely to admit an ED patient to inpatient status. **(** **Table 5** **)** After adjustment for patient-level variables, hospitals with an EDOU were more likely to disposition to observation (OR: 2.19, 9%%CI: 1.54–3.10) and more likely to disposition patients to inpatient admission (OR 1.26, 95%CI: 1.03–1.57). In adjusted analysis, in comparison to hospitals with no OUs, hospitals with HOUs were more likely to disposition patients to observation (OR: 1.91, 95%CI: 1.17–3.11) but not to inpatient admission. There was not a significant difference in the likelihood of disposition to observation or inpatient admission when comparing patients evaluated at hospitals with an EDOU versus hospitals with a HOU.

**Table 5:** Patient Disposition stratified by hospital Observation Unit presence, 2007–2008‡.

	Visits				Unadjusted OR Observation*		Adjusted OR Observation* †	
	n, % of all ED visits, (95%CI)				OR (95%CI)		OR (95%CI)	
Disposition	no OU	EDOU	HOU	Total	EDOU	HOU	EDOU	HOU
**Discharged**	120,912,000,	54,867,000,	19,966,000,	195,745,000,	Ref	Ref	Ref	Ref
	86.64%(85.30%–87.98%)	83.86% (81.90%–85.82)	84.02%(80.56%–87.48%)	85.57%(84.40%–87.53%)				
**Observation**	2,053,000,	1,709,000,	593,000,	4,355,000,	1.93 (1.27–2.60)	1.75 (1.11–2.76)	2.19 (1.54–3.10)	1.91 (1.17–3.11)
	1.47%(1.06%–1.88%)	2.61% (2.02%-3.21%)	2.50%(1.56%–3.43%)	1.90%(1.56%–3.86%)				
**Inpatient Admission**	16,587,000,	8,852,000,	3,204,000,	28,642,000,	1.17 (0.98–1.41)	1.17 (0.97–1.57)	1.26 (1.03–1.57)	1.08 (0.80–1.45)
	11.89% (10.65%–13.13%)	13.53% (11.77%–15.29%)	13.48%(10.39%–16.58%)	12.52%(11.48%–14.48%)				

‡All visit counts were rounded to the closest thousand, and all percentages are based on weighted frequencies prior to rounding.

*Odds Ratio reflect hospital level likelihood of disposition in comparison to hospitals with no OU.

†After adjustment for all patient level variables identified in table 4.

Several important limitations affect this analysis. First, several questions within NHAMCS changed or were not available for the entire survey period. The hospital survey introduced the questions about OUs in 2007, limiting the power of the analysis showing the lack of association between presence of an OU and use of observation services. Additionally, the wording of observation disposition changed slightly through the study period. In 2001 and 2002 observation disposition was worded as “admit for 23 hour observation.” In 2003 and 2004 this was changed to “admit to ED for observation.” From 2005 to 2008 this was changed to “admit to observation unit.” Changing the wording of the questions may account for some difference across years, but is unlikely to explain the large increase seen, as the wording was identical from 2005 to 2008, yet the proportion of ED visits undergoing observation increased from 0.88% to 1.87%. Second, we were unable to evaluate the care given during OU stay, as the NHAMCS is designed to study ED care and only includes data from ED charts and administrative records. Finally, NHAMCS is abstracted from ED charts and is limited by the quality of charting data. However, the NCHS has conducted NHAMCS since 1992 with robust quality control, such as training of office staff by Census field representatives and a two-way 10% independent verification procedure, leading to average keying error rate for non-medical items of less than 1% for items that required medical coding, discrepancy rates ranged below 1%.[13] It is possible that the understanding of observation disposition by chart abstracters has improved over time, and this may explain the increase in use of observation services.

## Discussion

This study presents nationally generalizable estimates of the number of dedicated OUs and the clinical range of observation use after ED visits. Our finding that over one third of EDs have dedicated OUs represents an increase from previous non-representative surveys.[12] In 2008, nearly 2% of all ED visits nationally were followed by observation care, a marked increase from several years prior. The 10 most frequent conditions undergoing observation evaluations include diagnoses for which there is strong evidence for the safety of observation care, such as chest pain syndromes, and some for which there is little evidence, such as abdominal pain. We identified patient characteristics associated with observation care, such as recent hospital discharge, highlighting the need to better understand the role of observation care among frequent ED users and in post-discharge care coordination. We also demonstrated an association between use of observation services and the presence of an OU at hospitals with either an EDOU or HOU.

We estimate that over one third of US hospitals providing emergency care have OUs. This appears to be an increase from a 2003 estimate derived from a non-representative survey of 522 hospitals, which reported that 19 percent of hospitals had a dedicated OU and an additional 12 percent planned to open an OU.[12] Additionally, we found that 52 percent of OUs are administratively controlled by the ED, which demonstrates that observation medicine in becoming a core competency of emergency medicine.

The hospital characteristics independently associated with the presence of an OU were ownership status, non-teaching status and ED length of visit. One potential explanation is that an OU can be a mitigating force against ED and inpatient crowding, and government and nonprofit hospitals are more crowded than proprietary hospitals creating a need for more robust observation services.[17] This suggests that financial considerations may be an important determinant of OU establishment, and worthy of further investigation of into the effect of different fiscal or governance structures on observation services. The association between OU presence and EDs with longer average length of visit may be similarly explained by the establishment of OUs as a response to overcrowded EDs or hospitals at full capacity.

This growth in observation use appears to cross a wide range of clinical condition. Not surprisingly, non-specific chest pain is the most common diagnostic group evaluated and treated with observation care, as it was the first and most studied observation condition.[6] Historically, many OUs were first designed as chest pain units based on early research that defined explicit inclusion and exclusion criteria for chest pain diagnostic pathways with clinical efficacy equivalent to inpatient admission.[18], [19] Interestingly, abdominal pain was the second most common RFV as well as CCS diagnosis evaluated in observation indicating widespread use of observation for a clinical condition that has not been formally studied. Clinical diagnoses that have a more robust evidence base for observation care such as syncope, congestive heart failure, and transient ischemic attack were all among the 10 most common observation conditions. These 10 diagnoses only account for around 40% of total observation care, and many diagnoses such as mood disorders, skin infections, and many unreported CCS categories (nearly 60% of all observation evaluations) represent the use of observation care outside of well established, validated pathways. This wide clinical application of observation reflects the relatively new state of observation medicine and either represents a deviation from validated clinical pathways or represents novel uses for observation care that are awaiting evaluation in rigorous comparative effectiveness studies.

The rapid growth in observation evaluation utilization between 2001 and 2008 has implications for healthcare delivery. Our data demonstrate a nearly fourfold increase in the use of observation care since 2001. The majority of this increase occurred between 2005 and 2007 for which there are several potential clinical, delivery system and policy explanations. Clinical reasons include the growing number of clinical conditions for which there are evidence based OU protocols, and the increasing age and medical complexity of the population.[6] In addition, delivery system changes such as hospital overcrowding, the development of observation care as a core emergency medicine practice, and increased ED care coordination services may also have contributed to increased utilization. As hospitals run close to or above occupancy, and there is little flexible capacity for unscheduled admissions from the ED, so observation services may be developed as an alternative to inpatient admission.[20] Finally, two policy changes may explain the growth in use of observation services. As aforementioned, in 2007 CMS expanded reimbursement for observation services from three conditions (chest pain, asthma and congestive heart failure) to any clinical condition, [11], a change soon modeled by private insurers that created a favorable reimbursement environment for observation. Additionally, in 2006, the Center for Medicare and Medicaid Services (CMS) was authorized to establish a Recovery Audit Contractor (RAC) process to retrospectively identify inappropriate use of Medicare and fine those providers and facilities that submitted inappropriate claims.[21] The RAC demonstration project was conducted in six states, and the primary mechanism for charge recovery identified was “inappropriate medical setting” charges for short inpatient stays creating a tremendous incentive for hospitals to manage patients with less severe illnesses in observation settings.[8]–[10] This trend is likely to continue as CMS begins public reporting and payment based on 30-day readmission, which will create an independent incentive to manage patients with congestive heart failure, recent acute myocardial infarction and pneumonia in the observation setting to avoid capture as a re-admission.[22]

We also found a considerable increase (over 6-fold) increase in admissions following observation evaluation between 2001 and 2008. The etiology of this increase is not well explained by this survey as the few records representing this disposition pathway (<2% of all inpatient admissions) do not provide sufficient power for analysis. This increase may be related to improved documentation of patient evaluation status as the general use of observation services grew, or may represent the use of observation services for patients with higher likelihoods of subsequent admission as a result of the healthcare delivery and policy pressures against inpatient admission described previously.

Several patient characteristics were associated with use of observation care compared to the ED discharge, but few were associated with use of observation compared to admission. Not surprisingly, markers of patient severity such as increasing age, arrival by ambulance and increasing triage severity were independently associated with use of observation care compared to ED discharge. Compared to inpatient admission, ED visit within the last 72 hours was associated with observation care which may reflect planned revisitation for conditions such as skin and soft tissue infection that have failed entirely outpatient therapy. Distinctly measuring scheduled versus unscheduled return to the ED will be important as healthcare organizations are held increasingly accountable for ED visitation and hospital re-admissions.

Finally, we demonstrated that OU presence, whether under ED control or not, was associated with increased use of observation evaluations after adjustment for patient characteristics. While this finding is not surprising, this study is not able to explain whether this use of observation services represents more efficient management of patients that would otherwise been dispositioned to inpatient services or overuse of observation services for patients that could have otherwise been discharged. Also, the association between hospitals with an EDOU (but not an HOU) and inpatient admission as the initial disposition was not expected given the prevailing notion that OU use can reduce inpatient admissions. This finding is not well-explained by this study, however, as we were unable to include multiple hospital-level variables or patient case-mix characteristics into the limited model. Understanding the appropriateness of observation disposition and the impact of observation use on inpatient admission is an important area for future work and will best be performed prospectively, to avoid the risk of hindsight bias.[23]

We present potential measures of OU utilization based on the use of observation in comparison to ED or inpatient care for top conditions and by year. The *ED observation proportion*, which highlights the relative use of observation for a clinical condition varied from less than one percent for injuries and cellulitis to 7.6% for transient ischemic attack. Similarly *admission from observation proportion* varied across clinical diagnoses indicating that some conditions like CHF (30% of observation patients subsequently admitted) are more prone to OU care failure and while conditions such as mood disorders and abdominal pain (10% and 14%, respectively) may represent clinical conditions with less diagnostic or treatment uncertainty and therefore prone to observation service overuse. Thus, the *observation to admission ratio* is a potential measure of OU efficiency that has been suggested by the Society for Chest Pain Centers and previously used to report variation in use of observation care for chest pain in Massachusetts.[24], [25] We found significant variation in this measure across clinical conditions which may be explained by efficient testing and treatment pathways for certain conditions such as chest pain (14%) or TIA (13%), while it is low for CHF (4%) indicating that current practices favor inpatient admission. The *observation to admission ratio* may be helpful for hospitals looking at the interaction between observation care and inpatient admission and help identify cases potentially at risk of RAC audit. Further study directed at understanding the impact of new public measures on observation care volume and efficiency will be critical.

### Conclusion

We report national estimates of observation unit presence and use based on patient and hospital level characteristics. About one in three EDs has an OU and the use of observation care following ED visits is rapidly increasing across a wide array of clinical conditions. These trends of increased observation care use in the setting of public policy initiatives directed at reducing inpatient admission highlight the importance of further study on the effect of observation care on the quality and efficiency of acute care delivery.
