# Correction: Use of Observation Care in US Emergency Departments, 2001 to 2008

**DOI:** 10.1371/annotation/e7927237-fcce-4738-9564-e9f5d85341cb

**Published:** 2012-01-18

**Authors:** Arjun K. Venkatesh, Benjamin P. Geisler, Jennifer J. Gibson Chambers, Christopher W. Baugh, J. Stephen Bohan, Jeremiah D. Schuur

There is an error in the funding statement. The correct funding is, “This work was conducted with support from Harvard Catalyst / The Harvard Clinical and Translational Science Center (NIH Award #UL1 RR 025758 and financial contributions from Harvard University and its affiliated academic health care centers). The content is solely the responsibility of the authors and does not necessarily represent the official views of Harvard Catalyst, Harvard University and its affiliated academic health care centers, the National Center for Research Resources, or the National Institutes of Health. In addition, the funders had no role in study design, data collection and analysis, decision to publish, or preparation of the manuscript.”

